# Fabrication of Polymeric Coatings with Controlled Microtopographies Using an Electrospraying Technique

**DOI:** 10.1371/journal.pone.0129960

**Published:** 2015-06-19

**Authors:** Qiongyu Guo, Jason P. Mather, Pine Yang, Mark Boden, Patrick T. Mather

**Affiliations:** 1 Department of Macromolecular Science and Engineering, Case Western Reserve University, Cleveland, Ohio, United States of America; 2 Syracuse Biomaterials Institute, Syracuse University, Syracuse, New York, United States of America; 3 Department of Biomedical and Chemical Engineering, Syracuse University, Syracuse, New York, United States of America; 4 Boston Scientific Corporation, Marlborough, Massachusetts, United States of America; North Carolina A&T State University, UNITED STATES

## Abstract

Surface topography of medical implants provides an important biophysical cue on guiding cellular functions at the cell-implant interface. However, few techniques are available to produce polymeric coatings with controlled microtopographies onto surgical implants, especially onto implant devices of small dimension and with complex structures such as drug-eluting stents. Therefore, the main objective of this study was to develop a new strategy to fabricate polymeric coatings using an electrospraying technique based on the uniqueness of this technique in that it can be used to produce a mist of charged droplets with a precise control of their shape and dimension. We hypothesized that this technique would allow facile manipulation of coating morphology by controlling the shape and dimension of electrosprayed droplets. More specifically, we employed the electrospraying technique to coat a layer of biodegradable polyurethane with tailored microtopographies onto commercial coronary stents. The topography of such stent coatings was modulated by controlling the ratio of round to stretched droplets or the ratio of round to crumped droplets under high electric field before deposition. The shape of electrosprayed droplets was governed by the stability of these charged droplets right after ejection or during their flight in the air. Using the electrospraying technique, we achieved conformal polymeric coatings with tailored microtopographies onto conductive surgical implants. The approach offers potential for controlling the surface topography of surgical implant devices to modulate their integration with surrounding tissues.

## Introduction

Surface topography of medical implants plays an important role in regulating cellular functions, including cell adhesion, migration, and differentiation, through guiding cell-implant interactions [1–4]. A variety of fabrication techniques have been employed to make microscale topographies with patterned or randomly distributed structures. For instance, classical processing methods, such as plasma spraying [5], acid etching [6], machining [7], and sandblasting [8], have been intensively studied to produce randomly roughened surfaces on metallic materials. However, very few techniques are available for producing polymeric coatings with controlled roughness or topology onto surgical implants, especially onto implant devices with small dimensions and complex structures.

Polymeric coatings have been widely applied to medical devices in order to improve the device performances in various aspects, including biocompatibility [9], biological functionalization [10], and controlled drug release [11–13]. For instance, drug-eluting stents (DES) have revolutionized percutaneous coronary intervention treatment by employing a thin layer of polymeric coating on the metallic stent struts for controlled drug release to reduce the rate of restenosis [14]. In the polymer-coated Taxus Paclitaxel-Eluting Stent (Boston Scientific, Natick, MA, USA), poly(styrene-*b*-isobutylene-*b*-styrene) (SIBS) triblock copolymers incorporated with the drug of paclitaxel are coated on the stent strut made by 316L stainless steel [15]. Nevertheless, the medical devices like DES invariably feature complicated architectures, presenting challenges for fabricating a polymeric coating on the devices with a controlled surface topography [13].

Recently, the electrospraying technique has received increasing attention for polymeric coating fabrication due to its facile controllability [16–19]. Electrospraying is an electrostatic processing method utilizing a high voltage under which a portion of a charged stationary liquid is ejected from the surface due to the electrical tension forces overcoming the surface tension force [20–23]. The charged liquid soon becomes unstable and breaks up into a mist of very fine charged droplets. The droplet size can be precisely controlled by electrospraying conditions with radii from hundreds of micrometers down to a few nanometers. Moreover, these droplets quickly dry in air and can undergo secondary breakup called Coulombic fission [24]. Here, electrostatic repulsion forces resulting from an increasing density of surface charges overcomes surface tension force. In addition, these charged droplets can target grounded conductive substrates, offering the potential to greatly increase the coating efficiency during conformal coating. Compared to conventional coating techniques, the electrospraying technique is uniquely suited as a coating technique for medical device processing given: (1) the ability to target a conductive substrate of the electrosprayed charged droplets, and (2) the ability to control topography of the coating surface through controlling droplet shape and dimension.

This study focuses on developing a new strategy to utilize electrospraying technique to fabricate uniform polymeric coatings with tailored microtopographies on Express coronary stents. Recently, we have developed a group of polyhedral oligosilsesquioxane (POSS)-based polymers featuring unique chemical, physical and mechanical properties for various applications [25–27]. A biodegradable POSS-based thermoplastic polyurethane (POSS TPU), which covalently incorporated POSS with poly(D, L-lactide) through urethane links, was employed in this work [28, 29]. This polyurethane coated on such stents has been demonstrated to feature a highly adjustable controllability on the release of paclitaxel [30]. In the present study, two electrospraying mechanisms were applied to control the microtopography of the polymeric coating on stent. Specifically, we tuned the electric field and flow rate of the polymer solution to manipulate either the primary breakup of the electrosprayed droplets right after ejection or the secondary breakup of the droplets during their flight in the air before deposition. The primary breakup of these droplets was controlled by the geometrical forms of the polymer solution jet and was utilized to tune the ratio of round to stretched droplets. The secondary breakup of the charged droplets was determined by droplet evaporation and charge density and was employed to tailor the ratio of round to crumped droplets. The microtopography of the stent coating was then modulated by adjusting the ratio of round to stretched droplets or the ratio of round to crumped droplets. In addition, a high coating efficiency was obtained due to the targeting capability of the electrosprayed droplets on the metallic stent.

## Materials and Methods

### Materials

Express Coronary Stents (16 mm length × 1.5 mm diameter) were kindly provided by Boston Scientific Corporation (Natick, MA, USA). The stent struts were smooth and made of 316L stainless steel. The mean roughness of the surface of bare metal stent is 4.3 ± 0.8 nm. The struts of the stents had a thickness of approximately 150 μm and width around 80 μm. Tetrahydrofuran (THF) and dimethylformamide (DMF) were purchased from Fisher and used as received. Polyhedral oligosilsesquioxane thermoplastic polyurethane (POSS TPU, ${\bar{M}}_{n}$ = 94.8 kg/mol, *T*_*g*_ = 38°C, *T*_*m*_ = 112°C, H = 1.70 J/g) was used. Synthesis of the POSS TPU was carried out as described earlier [28]. Briefly, this polymer was prepared by reacting a 12 kg/mol polyol (poly(D, L-lactide), PDLLA) with POSS diol using a lysine-derived diisocyanate (methyl 2,6-diisocyanatohexanoate, LDI) and typical urethane chemistry. The polyol was initiated by PEG ${\bar{M}}_{n}$ = 1 kg/mol and the mole feed ratio of POSS to polyol was 3.

### Fabrication of polymeric coatings using electrospraying

**Fig 1:**
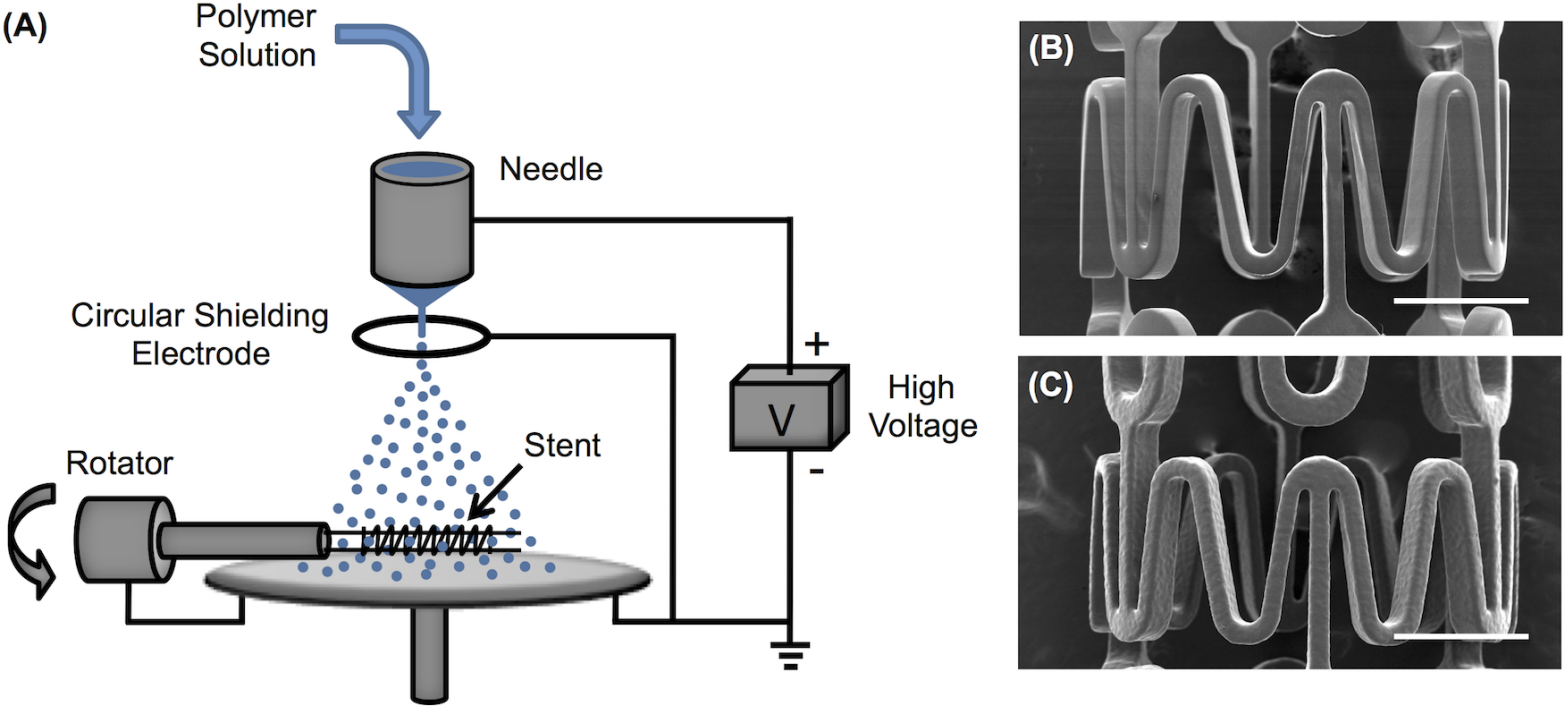
An electrospraying setup for stent-coating. (A) Schematic of an electrospraying setup for stent-coating using a circular shielding electrode placed right underneath the needle and above the aluminum plate. Continuous coating on Express coronary stent was achieved by exposed to the electrospraying mist for 30 min: (B) bare metal stent and (C) coated stent. Scale bar: 500 μm.

An electrospraying setup was designed to produce polymer droplets for fabricating polymeric coatings with tailored microtopographies on stents (Fig 1A). Specifically, a dilute polymer solution of 0.5 wt% POSS TPU/THF was utilized for electrospraying unless otherwise specified. A small percentage of DMF was added in THF in the polymer solution, i.e. 0.5 wt% POSS TPU/(THF:DMF = 95%:5%), to produce polymeric short fibers when controlling the microtopography of stent coating using different electrospraying modes as will be discussed below. A syringe pump (KD Scientific, Holliston, MA) was used to control the flow rate of the polymer solution in a 10 mL syringe (Hamilton, Reno, NV). A flow rate of 0.5 mL/h was applied unless otherwise specified. A programmable high voltage source (Ultravolt, Ronkonkoma, NY) was modulated by a DC power supply (Agilent E3630A, Newark, Chicago, IL). The positive electrode from the high voltage source was connected to the metal needle (304 stainless steel, Gauge 22, blunt needle point, outer diameter of 0.72 mm, and inner diameter of 0.41 mm). A circular aluminum plate (dia. 5 cm) was grounded and centered underneath the needle with a needle tip-to-plate distance of 5 cm unless otherwise specified. A fresh aluminum film was wrapped over the aluminum plate for each experiment to ensure good conductivity over the whole aluminum plate. In order to maintain stable droplet formation during electrospraying for some circumstances (indicated in the text below), a circular hoop-shaped shielding electrode (dia. 1.5 cm) made from a thin conductive wire (dia. 1 mm) was grounded and placed 0.5 cm below the needle. When used, the jet of droplets would traverse through the hoop toward the collector.

A stent rotation device was designed to support a stent using two stainless metal wires that spanned the length of each stent axially (Fig 1A). A needle tip-to-stent distance of 3.5 cm for stent coating by electrospraying was employed. The rotating stent supported on the two wires was grounded using a carbon brush dynamic contact. During stent coating, the stent was rotated at 20 rpm and inserted into the mist of electrosprayed droplets for a prescribed time up to 30 min, as detailed below.

### Electrosprayed droplet analysis

Electrosprayed droplets collected on a cover glass (22 mm x 22 mm, Thermo Scientific, Pittsburgh, PA) placed underneath the needle at distances from 3 cm up to 9 cm were examined by optical microscope (Olympus BX-51). Individual droplet sizes were determined using ImageJ (NIH) image analysis software. Deposited droplet size histograms were obtained on a total number of around 300 droplets at each condition.

### Stent coating efficiency assessment

The stent coating efficiency was measured using a polymer solution of 0.5 wt% POSS TPU/THF at a flow rate of 0.5 mL/h. The needle tip-to-stent distance was 3.5 cm, and the needle tip-to-plate distance was 5 cm. A circular electrode with diameter of 1.5 cm was placed 0.5 cm below the needle. An electric field of 6.8 kV/cm was employed. A bare metal stent rotating at 20 rpm was inserted into the mist of electrosprayed droplets for 30 min. The coated stent was left to dry at room temperature overnight. The stent mass before and after coating was measured three times using a Mettler-Toledo AX105 analytical balance (precision 0.01 mg, Columbus, OH). The average and standard deviation of coating efficiency were obtained based on six measurements acquired on different days.

### Scanning electron microscopy (SEM)

The morphologies of the polymeric coatings on stents were examined using scanning electron microscopy (SEM, Hitachi S4500) at an accelerating voltage of 6 kV after being coated with a 10 nm layer of Pd.

## Results and Discussion

### Modulation of droplet dimension using electric field

The electrospraying technique provides a convenient control to produce charged droplets with varying dimensions and geometries. This technique utilizes a high electric field to create very fine charged polymer droplets from an ejected polymer solution (Fig 1A). The ejected droplets travel rapidly towards a grounded aluminum plate located at some distance from the charged polymer solution under the influence of the electric field, and finally collect on the rotating stent inserted in the mist of the droplets, leading to a thin polymeric coating on the stent struts. As shown in Fig 1B and 1C, a layer of polymer was uniformly coated on an Express coronary stent after being exposed to the electrosprayed droplets for 30 min.

The ejected polymer solution near the needle tip forms a meniscus, which may adopt different geometrical forms depending on the stability of the polymer solution jet [31]. When electric stresses are balanced with the other forces existing in the meniscus of the polymer solution jet, including surface tension force, gravity and viscosity force, the liquid meniscus assumes a Taylor cone geometry [32] and undergoes Rayleigh’s capillary breakup [33]. This mode is called cone-jet mode and has been widely applied to produce polymer particles with monodispersed size-distribution [17, 34, 35]. When the forces on the liquid meniscus cannot be balanced under electrospraying conditions, the liquid meniscus becomes unstable and transforms to shapes distinct from the Taylor cone geometry [20].

**Fig 2:**
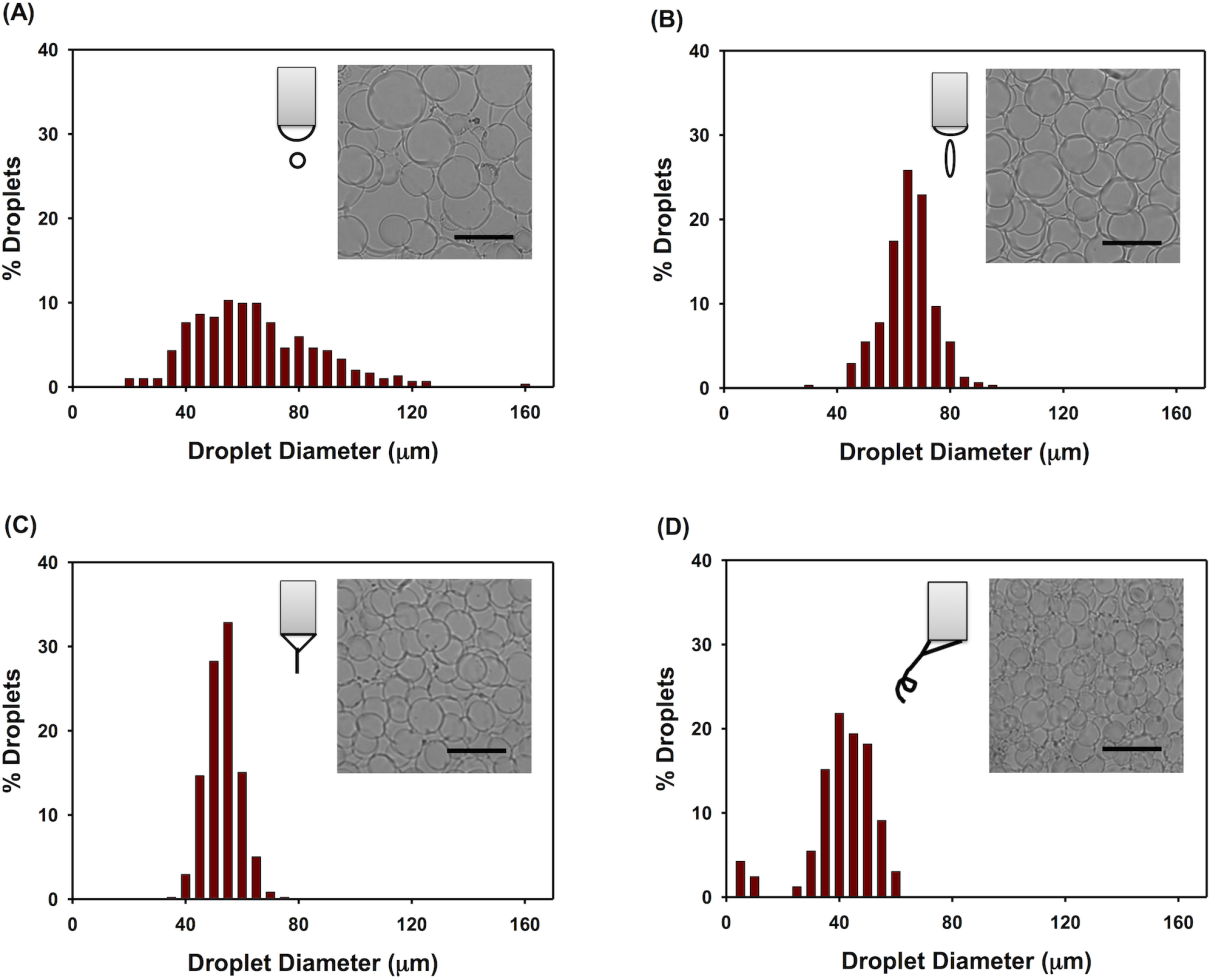
Deposited droplet size histogram obtained from different eletrospraying modes. Four eletrospraying modes were analyzed: (A) micro-dripping mode at electric field of 1.3 kV/cm, (B) spindle mode at electric field of 1.4 kV/cm, (C) cone-jet mode at electric field of 1.5 kV/cm, and (D) precession mode at electric field of 1.6 kV/cm. The optical microscopy images of the deposited droplets are shown in the inset Figs with scale bars of 100 μm.

The electric field magnitude plays a critical role in determining various electrospraying modes through controlling the meniscus formation of ejected polymer solution from the needle. When an electric field of 1.3 kV/cm was applied, the deposited droplets collected on a cover glass exhibited a diameter of 62 ± 22 μm with a very broad size distribution under a micro-dripping mode (Fig 2A). When the electric field was increased to 1.4 kV/cm, the electrospraying meniscus transformed to a spindle mode and the deposited droplets, compared to those under a micro-dripping mode, exhibited a diameter of 63 ± 9 μm with a narrowed size distribution (Fig 2B). As shown in Fig 2C, a cone-jet mode was obtained under the electric field of 1.5 kV/cm. The deposited droplets showed a smaller diameter of 51 ± 6 μm with a further narrowed size distribution. A precession mode was observed at 1.6 kV/cm, yielding deposited droplets with a bimodal size distribution (Fig 2D). One set of the droplets collected under the precession mode exhibited a diameter of 39 ± 12 μm, which is smaller than those under cone-jet mode. Another set of the deposited droplets with much smaller size (dia. < 10 μm) was also observed under the same precession mode. Therefore, the electrosprayed droplets coated on a substrate exhibited different shape and dimension under different electrospraying modes. Compared to the unstable electrospraying modes, the cone-jet mode was more controllable and produced droplets with dimensions of a narrower distribution. The geometries of the micro-dripping, spindle and precession modes are hemispherical, ellipsoidal and skewed cone, respectively.

### Modulation of droplet dimension by controlling flow rate

Under a cone-jet mode, the flow rate of polymer solution can be used to precisely adjust the size of electrosprayed droplets. In order to maintain a cone-jet mode under different flow rates, a circular shielding electrode was designed to create a strong electric field near the tip of the needle. This circular shielding electrode can stabilize the liquid meniscus coming out of the needle, especially for low conductivity solutions [21, 22]. As shown in Fig 1, a circular electrode with diameter of 1.5 cm placed 0.5 cm below the needle was found to be capable of efficiently stabilizing the cone-jet mode at 6.8 kV/cm during electrospraying for a wide range of flow rates.

**Fig 3:**
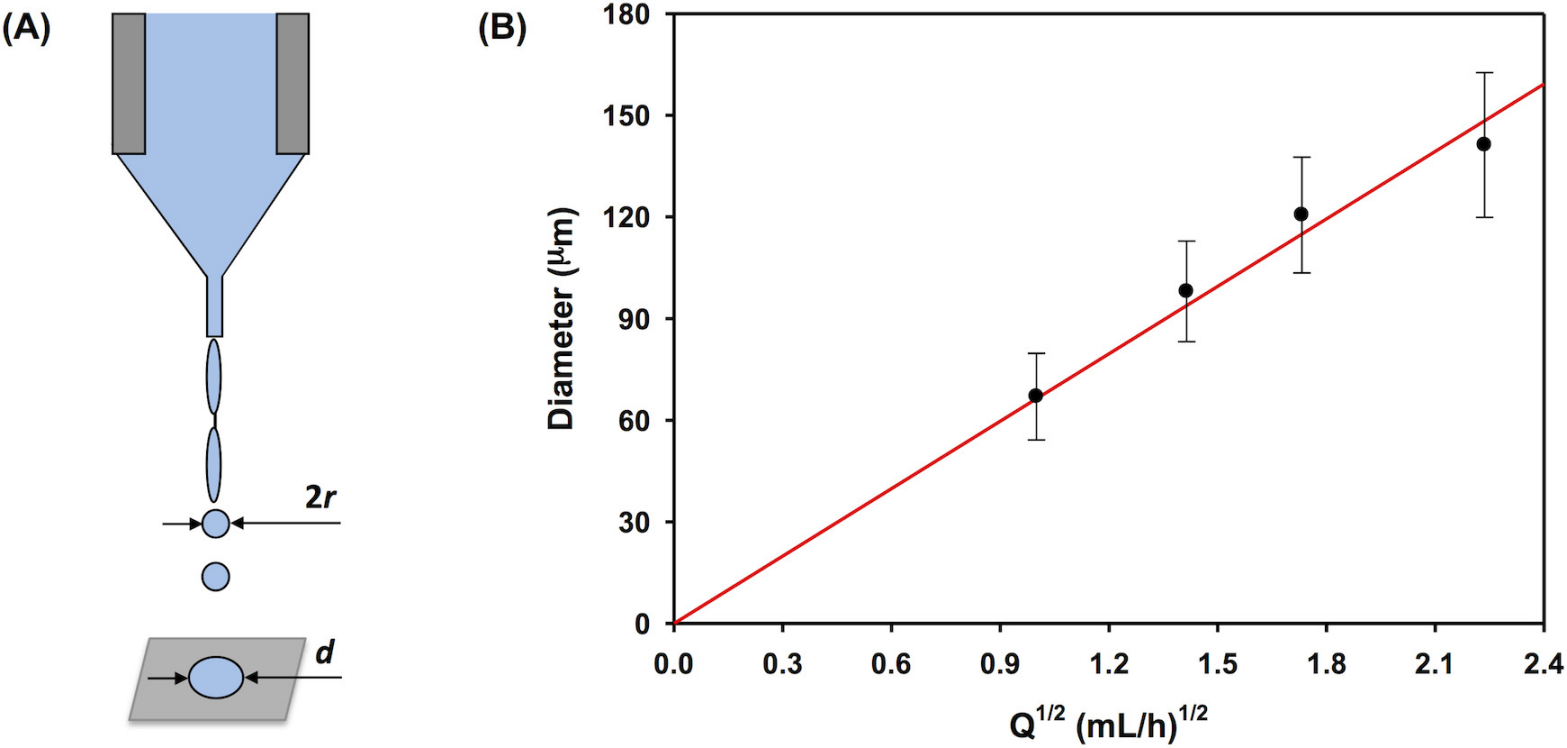
Formation of primary electrosprayed droplets. (A) Schematic diagram of the droplets formed from a jet through Rayleigh’s capillary breakup under a cone-jet mode during electrospraying process. (B) The diameter of deposited droplets vs. the square root of flow rate using the improved electrospraying setup with circular shielding electrode as shown in Fig 1A.

With the circular shielding electrode, we examined the impact of flow rate on the size of the deposited droplets while keeping the electric field constant at 6.8 kV/cm (Fig 3). For ideal liquids with low viscosity and low conductivity, the droplet size produced under cone-jet mode is expected to follow the relation [36],

$$r∼{\left[\varepsilon \varepsilon_{0}Q/K\right]}^{1/3}$$ (1)

where *r* is radius of primary droplet (Fig 3A), *ε* is dielectric constant of the liquid, *ε*_0_ is electrical permittivity of vacuum, *Q* is flow rate of the liquid, and *K* is electrical conductivity of the liquid. For a given polymer solution, its electrical permittivity and conductivity are both constant. Therefore, the flow rate is the only parameter available to control the size of electrosprayed droplets. If we assume the droplet deforms from a spherical shape to a cylindrical shape with relatively constant thickness, *h*, upon deposition, we can calculate the diameter of the deposited droplet, *d*, based on mass conservation,

$$d={\left(\frac{16}{3h}r^{3}\right)}^{1/2}∼r^{3/2}$$ (2)

then, we expect,

$$d∼Q^{1/2}$$ (3)

Indeed, as shown in Fig 3B, the diameter of the deposited droplets shows a linear relationship with *Q* ^1/2^, which confirms Eq (3) and applicability of Eq (1) to our conditions. Note that Fig 3B exhibited slightly decreased diameter of deposited droplets as compared to the estimated value at a flow rate beyond 1.8 (mL/h)^1/2^. This is probably caused by a Marangoni instability [37–39], wherein the deposited droplets are still fluidic and dewetting in the droplets is triggered by surface tension. This happens when the droplets are too large to effectively dry right after their deposition on the collector. We have observed similar effect on stent coatings, which will be discussed later.

### Modulation of droplet dimension by controlling droplet breakup

**Fig 4:**
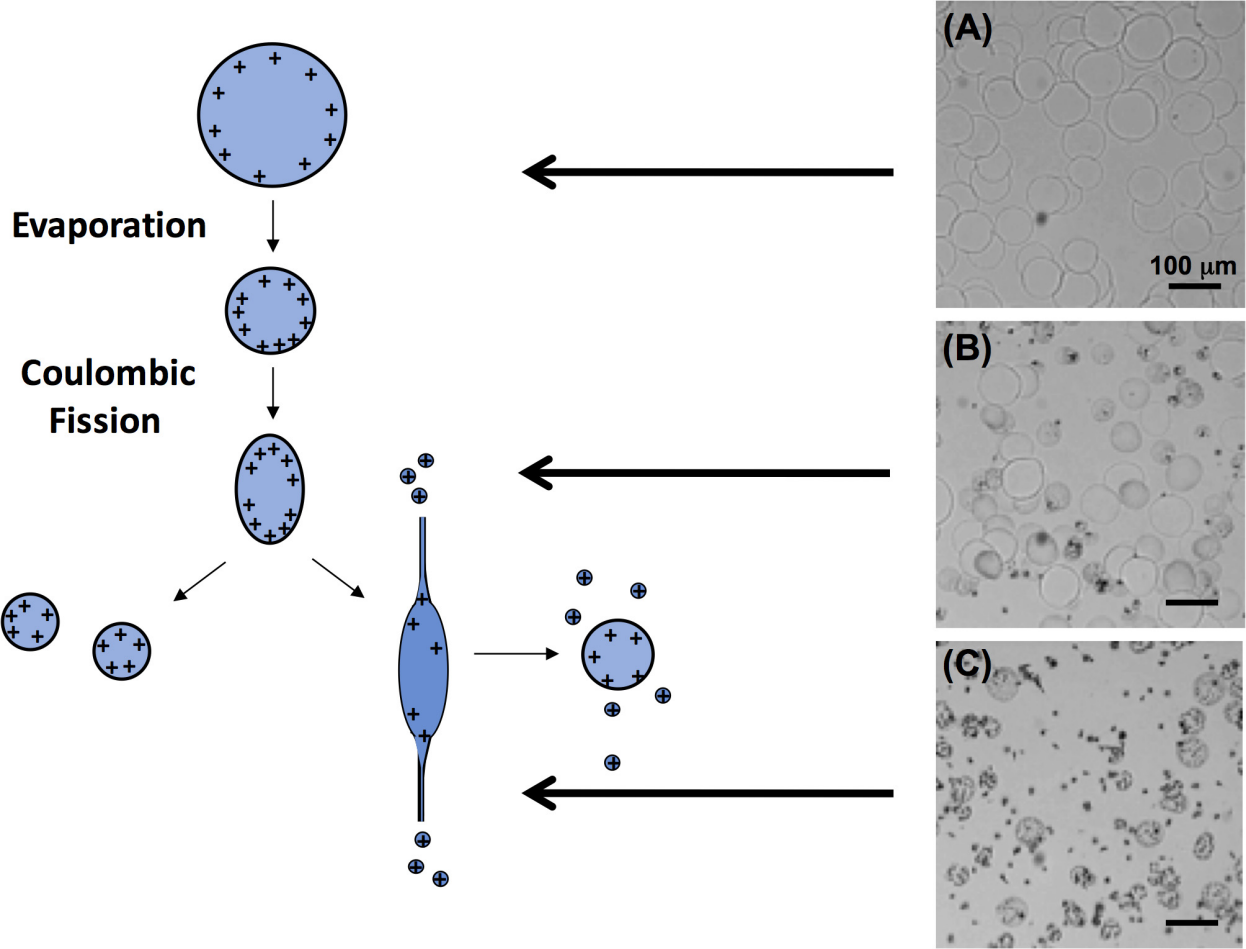
Breakup of electrosprayed droplets due to Coulombic fission. Schematic (left) and optical microscopy (right) images of deposited droplets collected from electrspraying mist at different tip-to-collector distance of (A) 3 cm, (B) 6 cm and (C) 9 cm, using a same scale bar of 200 μm. Fragmented droplets were formed in (B) and (C) due to Coulombic fission. The electrospraying process was performed using a constant tip-to-plate distance of 9 cm.

A unique phenomenon of charged polymer droplets produced from electrospraying is Coulombic fission: droplet breakup that occurs when electrostatic repulsion forces resulting from surface charge increase beyond the surface tension force of the droplets [24]. This happens in transporting electrosprayed droplets before they are collected due to the evaporation of the solvent from the droplets, which increasingly concentrates surface charge. In order to test for the Coulombic fission phenomenon, we placed the grounded aluminum plate 9 cm underneath the needle, employed a circular shielding electrode 0.5 cm below the needle, and collected the electrosprayed droplets at different distances from the needle using a cover glass. As shown in Fig 4, the electrosprayed droplets collected on the cover glass at different distances from the tip exhibited different morphologies. For a tip-to-collector distance of 3 cm, droplets with round edges were obtained, which indicates that Coulombic fission had not occurred in these droplets. When the tip-to-collector distance was increased to 6 cm, a fraction of the droplets broke into small pieces with ragged morphology. When the tip-to-collector distance increased further to 9 cm, only small, fragmented droplets with ragged morphology were obtained. Moreover, these droplets showed two quite distinct dimensions resulting from the breakup of the primary droplets due to Coulombic fission.

### Targeted coating on metallic stents by electrospraying

The positively charged electrosprayed droplets are unique in that they can target the grounded metallic stent and produce a continuous coating on the stent with a high coating efficiency. Here, the coating efficiency is calculated by,

$$\text{Coating efficiency}=\frac{\text{Coating mass on the stent}}{\text{Total mass of sprayed polymer}}\times 100\%$$ (4)

When a cone-jet mode was applied with the assistance of the ring electrode, the coating efficiency was controlled by the electrospraying setting, including the tip-to-stent distance and tip-to-plate distance. During electrospraying, the droplet spray area, which was mainly determined by the repulsion and gravity forces of the charged electrosprayed droplets, was influenced by the tip-to-plate distance and tip-to-stent distance. When a tip-to-stent distance of 3.5 cm and tip-to-plate distance of 5 cm were applied, a spray area with diameter of 3 cm was observed and completely covered the stent (1.6 cm in length× 1.5 mm in diameter) fixed in space longitudinally. A further increase of the spray area by increasing the tip-to-plate distance would lead to a decrease of the coating efficiency, whereas a decrease of the spray area could lead to uneven coating along the stent. When the spray area was fixed at 3 cm, the coating efficiency was found to be 31±5%. If we assume that the electrosprayed droplets would be deposited in the spray area uniformly, the coating efficiency would equal the ratio of the area of the stent to the spray area at the same horizontal plane. This resulted in an estimated coating efficiency as low as 3%, which is a ten-fold lower than the coating efficiency we obtained. This indicates that the charged droplets produced from electrospraying targeted the grounded stent, leading to a dramatically increased coating efficiency.

### Microtopography control of stent coating by electrospraying modes

**Fig 5:**
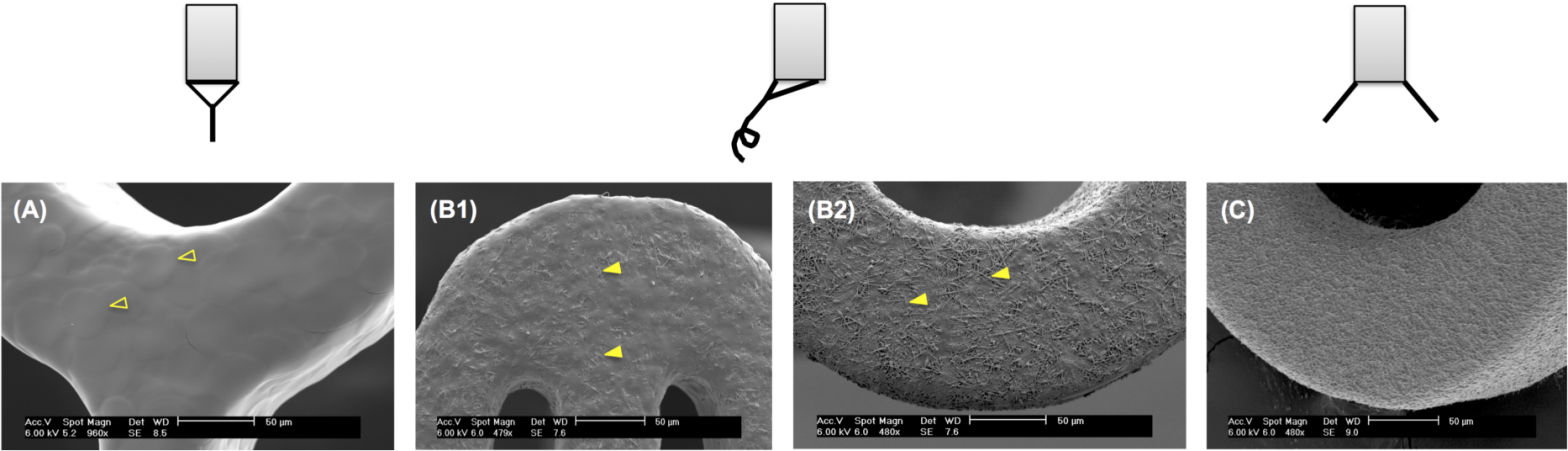
Coating morphology obtained at different electrospraying modes. SEM images of Express coronary stents coated by electrosprayed droplets obtained at different electrospraying modes: (A) cone-jet mode, (B) precession mode, and (C) multi-jet mode. These electrospraying modes were achieved at an increasing electric field of (A) 1.5 kV/cm, (B1) 1.6 kV/cm, (B2) 1.7 kV/cm, and (C) 1.8 kV/cm, respectively. Round and stretched deposited droplets were highlighted in empty yellow triangles (A) and solid yellow triangles (B), respectively.

Stent coatings with controlled microtopographies were obtained from electrosprayed droplets produced using different electrospraying modes, as shown in Fig 5. Continuous stent coatings with smooth topography were obtained when the deposited droplets (dia. 30–60 μm) were formed using a cone-jet mode obtained at an electric field of 1.5 kV/cm (Fig 5A). A precession mode was derived at 1.6 kV/cm and 1.7 kV/cm, separately. As shown in Fig 5B, a combination of round and stretched droplets were produced and simultaneously coated on the stent, resulting in a layer of roughened polymeric coating. The stretched droplets appear as short fibers due to their high aspect ratios. Importantly, compared to the stent coating obtained at 1.6 kV/cm (Fig 5B1), the increased electric field of 1.7 kV/cm (Fig 5B2) produced a higher ratio of stretched to round droplets and higher aspect ratio of the stretched droplets in the coating. Under a multi-jet mode at 1.8 kV/cm, only stretched droplets were formed and deposited on the stent surface, yielding a fibrous structure on the stent coating (Fig 5C). These short fibers deposited on stent are distinct from conventional electrospun nanofibers with an infinite aspect ratio [40], which are unable to continuously coat the stent surface without covering the empty spaces between stent struts.

**Fig 6:**
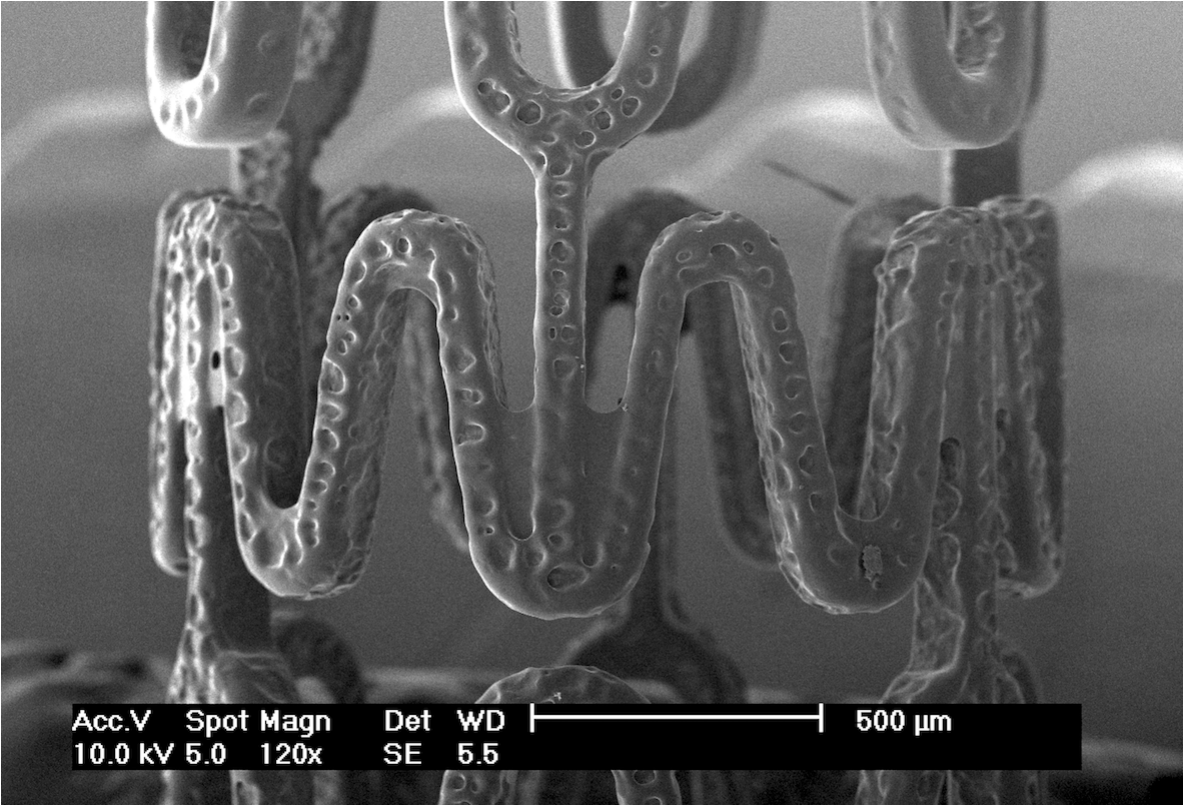
SEM image of a stent coated with large electrosprayed droplets (dia. ~120 μm). A non-uniform coating with large pores was observed on the stent due to dewetting of the liquid coating caused by ineffective evaporation and solidification of the deposited droplets during coating process.

We observed that the morphology of electrosprayed droplets coated on stent surfaces was slightly different from those deposited on glass slide. This may be explained by the change of local electric field around the stent resulting from insertion of the stent in the coating apparatus, itself. In addition, electrosprayed droplets too large in size (dia. >100 μm) are not suitable for stent coating due to the small dimension of each stent strut (width ~150 μm). The polymer droplets could specifically accumulate along the edge and across the corner of the stent struts, leading to non-uniform coatings with large pores on the stent (Fig 6). This phenomenon relates to the Marangoni instability [37–39], wherein dewetting of liquid coatings is caused by surface tension gradients. This is applicable in our process when the coating is still fluid after the deposition of the electrosprayed droplets on the stent.

### Microtopography control of stent coating by Coulombic fission

**Fig 7:**
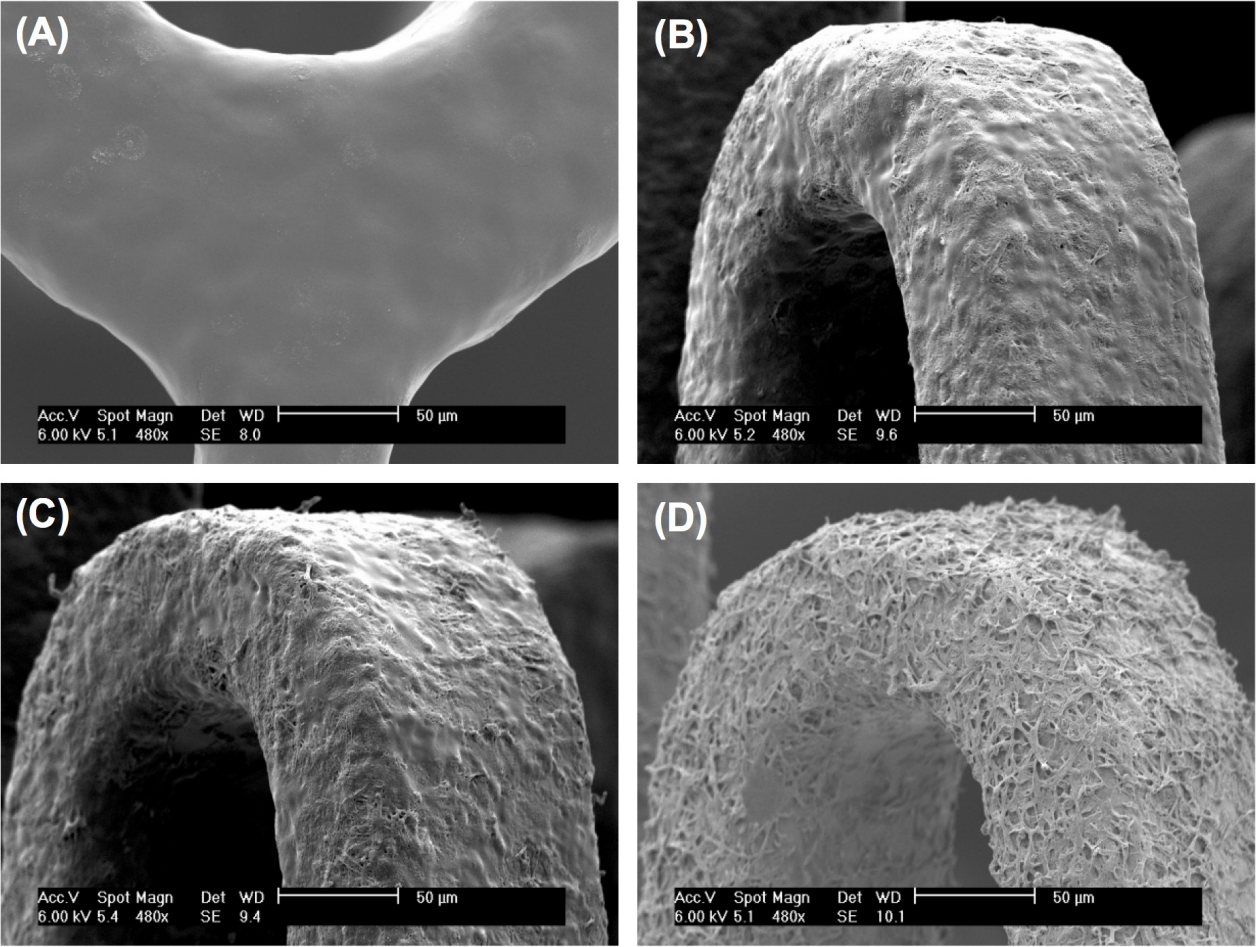
Coating morphology controlled by Coulombic fission. SEM images of Express coronary stents coated by electrosprayed droplets obtained at different flow rates with varying degree of Coulombic fission: (A) 0.5 mL/h, (B) 0.4 mL/h, (C) 0.3 mL/h, and (D) 0.2 mL/h.

The adjustment of the microtopographies of stent coatings was accomplished by changing the ratio of crumpled droplets to smooth droplets formed by Coulombic fission using electrospraying technique under a cone-jet mode stabilized by a circular shielding electrode. As shown in Fig 7, the roughness of stent coatings increased as the volumetric flow rate, *Q*, decreased gradually from 0.5 mL/h to 0.2 mL/h. Fig 7A, with *Q* = 0.5 mL/h, reveals a smooth surface morphology, whereas Fig 7D, while *Q* = 0.2 mL/h results in a web-like coating on the stent. This could be attributed to the increasing instability of electrosprayed droplets at decreasing flow rate. As we discussed previously, the electrosprayed droplets become unstable and undergo Coulombic fission to be deformed or split when their electrostatic repulsion increased beyond the surface tension during their transport in the air before collected. The smaller the droplets produced from the Taylor cone, the higher surface-to-volume ratio of the droplets, and the higher the evaporation rate of the solvent in the droplets. The result is less stable droplets. Therefore, as we decreased the solution flow rate, the size of the electrosprayed droplets also reduced, leading to more unstable and rough droplets collected on the stent. Fig 6B and 6C obtained for coatings produced at flow rates of 0.4 mL/h and 0.3 mL/h, respectively, clearly exhibit both of the crumpled droplets and smooth droplets on the stent coatings. All stent coatings shown in Figs 5–7 feature uniform surface topography in the whole stent.

## Conclusions

In this study, we presented a new strategy to employ the electrospraying technique to fabricate polymeric coatings with varying microtopographies on coronary stents. We systematically investigated tuning electrospraying processing conditions, including electric field, flow rate and tip-to-collector distance, to manipulate the primary breakup and secondary breakup of the electrosprayed droplets for stent coatings. Different mechanisms of the electrospraying technique were employed to precisely control the droplet formation, droplet dimension and droplet breakup. Smooth stent coatings were achieved using the proper droplet size between 30 to 60 μm under cone-jet mode before Coulombic fission happens. The microtopography of stent coating was varied conveniently by the electrospraying technique utilizing different electrospraying modes with or without Coulombic fission. Drugs can also be easily incorporated into the polymeric coating by electrospraying polymer solutions containing dissolved drugs [30, 41]. Therefore, the electrospraying technique was proven to be suitable to produce coating on electrically conductive surgical implants, and can be argued to be superior to both dip coating and air-brush spraying techniques in terms of surface topography control and coating efficiency. Enhancing stent coating surface topography can potentially modulate surface properties such as surface energy and wettability, which directly impact protein absorption and cell responses at the interface between the implant and host tissue. The great potential of this electrospraying technique has been only evaluated in limited research explorations, but undoubtedly deserves deeper attention in various biomedical applications from a small scale of microstructured particles to a large scale of bioscaffolds, and from pre-defined processing to in-situ custom-designed treatments.

## Supporting Information

S1 Data Deposited droplet size distribution data represented in Fig 2. (XLSX)

S1 Table P value comparison of deposited droplet diameters obtained from different eletrospraying modes represented in Fig 2. (XLSX)
